# *Haemophilus influenzae* Type b Reemergence after Combination Immunization

**DOI:** 10.3201/eid1206.051451

**Published:** 2006-06

**Authors:** Nik G. Johnson, Jens U. Ruggeberg, Gail F. Balfour, Y. Chen Lee, Helen Liddy, Diane Irving, Joanna Sheldon, Mary P.E. Slack, Andrew J. Pollard, Paul T. Heath

**Affiliations:** * St George's University of London, London, United Kingdom;; † University of Oxford, Oxford, United Kingdom;; ‡ St. George's Hospital, London, United Kingdom;; § Health Protection Agency Centre for Infections, London, United Kingdom

**Keywords:** Haemophilus influenzae type b, haemophilus vaccines, antibody avidity, diphtheria-tetanus-acellular pertussis vaccines, antibodies, carrier state/epidemiology, research

## Abstract

An increase in *Haemophilus influenzae* type b (Hib) in British children has been linked to the widespread use of a diphtheria/tetanus/acellular pertussis combination vaccine (DTaP-Hib). We measured anti-polyribosyl-ribitol phosphate antibody concentration and avidity before and after a Hib booster in 176 children 2–4 years of age who had received 3 doses of DTP-Hib (either DT whole cell pertussis-Hib or DTaP-Hib) combination vaccine in infancy. We also measured pharyngeal carriage of Hib. Antibody concentrations before and avidity indices after vaccination were low (geometric mean concentration 0.46 μg/mL, 95% confidence interval [CI] 0.36–0.58; geometric mean avidity index 0.16, 95% CI 0.14–0.18) and inversely related to the number of previous doses of DTaP-Hib (p = 0.02 and p<0.001, respectively). Hib was found in 2.1% (95% CI 0.7%–6.0%) of study participants. Our data support an association between DTaP-Hib vaccine combinations and clinical Hib disease through an effect on antibody concentration and avidity.

## Abstract 2

Combination vaccines may suppress Hib antibody concentration and avidity.

After the introduction of conjugate *Haemophilus influenzae* type b (Hib) vaccines in October 1992, the incidence of invasive Hib disease in England and Wales dramatically declined. From 1990 to 1992, the annual incidence in children <5 years of age was 20.5–22.9 per 100,000 and by 1998 it had fallen to 0.65 per 100,000 (*1*). However, since 1999 the number of invasive Hib infections has risen, with an increase every year in the number of cases in children born from 1996 to 2001; by 2002 the disease incidence had reached 4.58 per 100,000 (*1*). This rise coincided with a temporary change in the type of Hib vaccine combinations given for primary immunization. An acellular pertussis combination vaccine (DTaP-Hib) was used from 1999 to 2002 because of a shortage of the whole cell pertussis combination (DTwP-Hib) vaccine. It also coincided with the introduction of routine immunization with meningococcal conjugate (MCC) vaccines in 1999.

Most, but not all (*2*), DTaP-Hib vaccines result in lower Hib antibody concentrations shortly after vaccination when compared with DTwP-Hib vaccines (*3*), but the persistence of this effect has not been studied and its clinical significance is controversial (*3*). However, a UK case control study has demonstrated an increased risk for invasive Hib disease in children who received >2 doses of DTaP-Hib, which suggests that this effect may be clinically relevant (*4*). Suppression of Hib antibody responses by other concomitantly administered vaccines has also been described. These include pneumococcal conjugate vaccines (*5*), inactivated polio vaccines (*6*), and MCC vaccines (*7*), even when given by separate injection. With respect to MCC vaccines, lower Hib responses were observed when the CRM-197 conjugate MCC vaccine was administered simultaneously as compared with the tetanus toxoid conjugate MCC vaccine (*7*).

In response to the rising incidence of Hib disease, a national booster campaign was initiated in 2003 in which an extra dose of Hib vaccine was offered for all children between 6 months and 4 years of age. We used this opportunity to assess whether the number of DTaP-Hib vaccines given during primary immunization was related to the serum concentration and avidity of Hib antibody both before and after the booster in children 2–4 years of age. We also aimed to assess the prevalence of pharyngeal Hib carriage in this age group.

## Methods

The UK primary vaccine schedule consisted of 3 doses of Hib conjugate vaccine, DTP vaccine, oral polio vaccine, and MCC vaccine all administered at 2, 3, and 4 months of age. For their primary infant schedule, the participants in this study received either a DTwP-Hib (ACT-HIB DTP, Pasteur Merieux MSD Ltd, Maidenhead, UK) or DTaP-Hib (Infanrix-Hib, GlaxoSmithKline, Middlesex, UK). The Hib conjugate in both consisted of PRP conjugated to tetanus toxoid (PRP-T), and the acellular pertussis vaccine contained 3 pertussis components. The participants in the study may also have received 1 of 2 different meningococcal conjugate vaccines, a CRM197-based conjugate or a tetanus toxoid–based conjugate.

The names of children 2–4 years of age with full Hib vaccination (3 doses) were obtained from district computerized immunization records. After written informed parental consent was obtained, pharyngeal swabs and blood samples were obtained from the children, and doses of Hib conjugate vaccine were administered. Four to 6 weeks later, a second blood sample was obtained from each child. Dates and types of previous vaccinations were obtained from immunization records and the children's handheld record. We were particularly interested in the types of DTP-Hib and MCC vaccines the children had received. The study was approved by the Wandsworth Local Research ethics committee (reference 00.6.14).

We used an enzyme-linked immunosorbent assay (ELISA) method to measure immunoglobulin G antibodies to PRP. The minimum level of detection of the assay was 0.11 μg/mL, and values <0.11 μg/mL were recorded as 0.05 μg/mL for summary calculations. Anti-PRP concentrations were log transformed, and the geometric mean concentration (GMC) and 95% confidence intervals (CI) were calculated. Avidity was determined by using a thiocyanate elution ELISA (*8*) and calculating the log-transformed data as the geometric mean avidity index. Throat swab specimens were obtained with a cotton-tipped swab, placed in transport media, and cultured on anti-serum agar. Strains identified as *H. influenzae* were analyzed by conventional slide agglutination and polymerase chain reaction (*9*).

We compared proportions by using Fisher exact test and GMCs between groups by using Mann-Whitney or Kruskal-Wallis tests. Linear regression was used to explore the effects of several variables on anti-PRP antibody concentration. The variables included in the model were age, number of DTaP-Hib vaccines, and type of MCC vaccines received, and for the post-booster anti-PRP concentration, the time between vaccination and blood sampling. For statistical analysis, we used SPSS, version 12.0 (SPSS Inc., Chicago, IL, USA) for Windows (Microsoft Corp., Redmond, WA, USA).

## Results

We recruited 195 study participants from April 2003 to January 2004. Throat swabs were taken from 143 participants, and blood samples were taken from 176 participants. Their median age was 37.8 months (range 24–50 months), and 92% had received their third primary vaccine dose by 7 months of age.

We identified the type of DTP-Hib combination vaccine used in all 3 primary vaccinations in 163 (92.6%) participants and the number of doses and type of MCC vaccine in 159 (90.3%) participants. Participants were assigned to 1 of 4 groups based on the number of acellular pertussis vaccines (DTaP-Hib) received (0, 1, 2, or 3 doses). In 10 additional participants, 2 of the 3 vaccine combinations they had received were classified. Type of combination vaccine received varied according to participant age with younger children more likely to have received an acellular pertussis combination. The median age of participants who received 2 or 3 doses of DTaP-Hib was 32.4 months versus 40.9 months for those who received 2 or 3 doses of DTwP-Hib (p = 0.001).

### Prebooster Anti-PRP Antibody Concentrations

**Figure 1:**
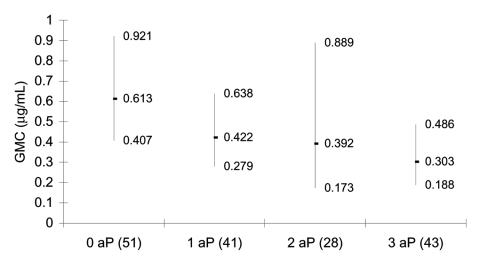
Geometric mean anti-polyribosyl-ribitol phosphate antibody concentration before booster (95% confidence intervals) in 2- to 4-year-old children, according to number of doses of acellular pertussis (aP) containing *Haemophilus influenzae* type b combination vaccines received in infancy. Number of participants is shown in parentheses. GMC, geometric mean concentration.

**Figure 2:**
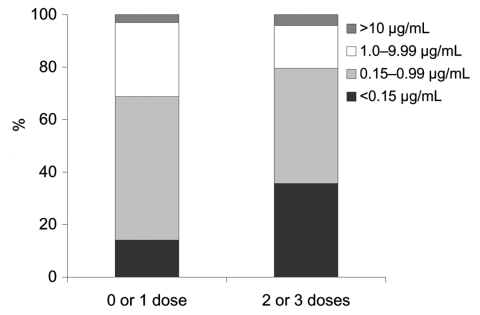
Anti-polyribosyl-ribitol phosphate antibody concentrations in 2- to 4-year-old children, according to number of doses of acellular pertussis containing *Haemophilus influenzae* type b combination vaccines received in infancy. Proportion achieving different concentrations is shown.

The prebooster GMC for all participants was 0.46 μg/mL (95% CI 0.36–0.58) with 40 of 175 (22.9%) <0.15 μg/mL and 128 (73.1%) <1.0 μg/mL. For those who received all 3 doses as DTwP-Hib, the GMC was 0.61 μg/mL (95% CI 0.41–0.92); for those who received all 3 doses as DTaP-Hib, the GMC was 0.30 μg/mL (0.19–0.49). There was a significant trend in prebooster GMC according to the number of doses of acellular pertussis vaccine received, with increasing doses of DTaP-Hib associated with decreasing anti-PRP antibody concentrations (p = 0.02) (Figure 1). Receiving 2 or 3 doses of DTaP-Hib was associated with a higher proportion of children with a nonprotective concentration (<0.15 μg/mL) than receiving 2 or 3 doses of DTwP-Hib (36% vs. 14%, p = 0.002) (Figure 2). On linear regression analysis, only the type of DTP-Hib vaccine combination received (p = 0.034) was associated with prebooster antibody concentration.

With regard to MCC vaccines, 91% of children received the CRM197 containing conjugate vaccines, and 9% received the tetanus toxoid conjugate vaccine. No significant differences were found between anti-PRP antibody concentrations achieved according to type of MCC vaccine received (data not shown).

### Postbooster Anti-PRP Antibody Concentrations

The postbooster GMC was 156.1 μg/mL (95% CI 133.5–182.4); 1 of 170 (0.6%) <1.0 μg/mL; 168 (99%) >10 μg/mL; median fold rise 439, range 0.9–9,200), obtained at a median of 31 days (range 26–64). The GMC was 153.1 μg/mL (113.5–207.0, n = 50) for those who had received all primary 3 doses as DTwP-Hib; 179.1 μg/mL (139.6–229.6, n = 38) for those who received 2 doses of DTwP-Hib and 1 dose of DTaP-Hib; 147.6 μg/mL (87.1–249.5, n = 27) for those who received 1 dose DTwP-Hib and 2 doses DTaP-Hib; and 134.0 μg/mL (96.4–186.2, n = 42) for those who received all 3 doses as DTaP-Hib. None of the variables included in the model was associated with postbooster anti-PRP antibody concentration.

### Anti-PRP Avidity

**Figure 3:**
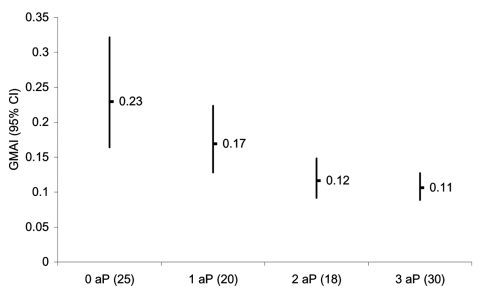
Geometric mean avidity index (GMAI) (95% confidence intervals [CI]) after booster in 2- to 4-year-old children, according to number of doses of acellular pertussis containing *Haemophilus influenzae* type b combination vaccines received in infancy. Number of participants is shown in parentheses.

No significant differences in geometric mean avidity index were found before and after receiving the Hib booster vaccine (data not shown). A significant inverse trend to lower postbooster avidity levels was evident according to the number of doses of DTaP-Hib received (p<0.001) (Figure 3).

### Pharyngeal Hib Carriage

Three of 143 participants (2.1%, 95% CI 0.7%–6.0%) were found to be carrying Hib on pharyngeal culture. One child had received all DTwP-Hib, and the other 2 had received all DTaP-Hib vaccines. The prebooster anti-PRP antibody concentrations in the 3 carriers were high: 63.9, 123.7, and 4.2 μg/mL. An additional 9 participants had prebooster anti-PRP antibody concentrations >5μg/mL (4 participants had concentrations >10 μg/mL), which suggests recent or current carriage of Hib or of a cross-reactive antigen.

## Discussion

We have shown that Hib antibody concentrations in healthy UK children 2–4 years of age were low in 2003, with 23% of children unprotected based on a serologic correlate of 0.15 μg/mL and 73% of children unprotected based on a correlate of 1.0 μg/mL. This finding is consistent with national serologic data from 2000, which also showed that median anti-PRP antibody concentrations from children 2–4 years of age in 2000 were significantly lower than those from 1994 (*10*). The probable explanation is that children in this age group in 1994 had received 1 dose of Hib conjugate vaccine after 12 months of age as part of a catch-up program, while similarly aged children in 2000 and 2003 had received vaccine before 6 months of age as part of primary immunization. In the absence of vaccine boosting, anti-PRP antibody levels induced by vaccination in infancy wane over 2–3 years (*11*). However, we have shown a significant and lasting effect on anti-PRP antibody levels of the type of DTP-Hib combination vaccine used for primary vaccination. Participants who received all 3 primary doses as DTaP-Hib had antibody concentrations 2–4 years later that were approximately half those of participants who received all 3 primary doses as DTwP-Hib. This extends the findings of earlier studies in which Hib antibody concentrations were measured shortly after vaccination and found to be lower in recipients of DTaP-Hib vaccines (*3*).

Our data on avidity contrast with those of others who found that avidity appeared unaffected by receipt of acellular pertussis–containing Hib combination vaccines in the short term (to 1 year of age) (*12**,**13*). Our data suggest that DTaP can interfere with the normal antibody avidity maturation that occurs after priming with Hib vaccine. Since the avidity of serum antibody is likely to relate to the functional activity of the serum (*14*), the combination of decreased antibody concentration and decreased avidity suggests an increased susceptibility to Hib disease. This occurrence may provide the biological basis for the finding of an elevated risk of clinical vaccine failure in recipients of >2 doses of DTaP-Hib in a recent UK case control study (*4*).

Nearly all vaccinees responded well to the Hib booster dose, with 95% having >10-fold rises in antibody concentrations (median rise 439-fold) and 99% achieving concentrations >10 μg/mL. This result was independent of which combination vaccine they received in infancy. For most of the participants, the magnitude of their response is consistent with a memory response and implies that acellular pertussis–containing Hib combination vaccines do not impair immune memory. This finding was also demonstrated in earlier studies and formed the basis for the argument that suppression of Hib antibody concentrations by acellular pertussis–containing vaccines is not clinically relevant (*3**,**13**,**15*). Our data indicate, however, that the presence of memory, as judged by antibody response to a booster dose, is not necessarily a reliable surrogate of clinical protection. This conclusion is supported by the observation that many Hib-vaccinated children in whom clinical Hib disease later develops have a better convalescent-phase antibody response than unvaccinated children in whom Hib disease develops (*16*). In the absence of circulating antibody of sufficient quantity and quality, once Hib is encountered, the memory antibody response may be too slow to prevent invasion occurring.

The occurrence of cases of Hib disease implies ongoing transmission of Hib in the population, and our finding of a pharyngeal carriage rate of 2.1% implies that Hib was circulating in this susceptible age group. Significantly elevated antibody concentrations (>5 μg/mL) found in 9 additional participants suggests that this carriage rate will likely be a minimum estimate. Another UK study found no Hib carriage in similarly aged children in 1997 and 2002 (*17*). This difference may be explained by the population sampled or the consistent use of anti-serum agar in our study.

Reemergence of invasive Hib disease in a well-vaccinated population in Alaska in 1997 was attributed to ongoing carriage of Hib in the context of a low prevalence of Hib antibody because of a change of Hib conjugate vaccine (*18*). Similarly in the United Kingdom, the combination of low anti-PRP antibody concentration and quality, together with the presence of Hib carriage, likely explains the recent resurgence of invasive Hib disease. Low antibody concentrations in UK children 2–4 year of age is primarily due to the waning of vaccine-induced antibody after infant vaccination without a routine booster dose. This result may be a particular issue where accelerated schedules, such as the UK 2,3,4-month schedule, are employed because such schedules may be less immunogenic when compared to extended schedules (*19*). Our study indicates that this phenomenon has been compounded by the use of acellular pertussis Hib combination vaccines with lower Hib immunogenicity. As with the Alaskan experience, these results emphasize the importance of long-term surveillance of vaccine preventable diseases, particularly during changes to routine immunization schedules.
